# An enigmatic translocation of the vertebrate primordial eye field

**DOI:** 10.1186/s12862-020-01693-6

**Published:** 2020-10-02

**Authors:** R. G. Loosemore, S. D. Matthaei, T. C. Stanger

**Affiliations:** 1 Maclean District Hospital, Union St, Maclean, NSW 2463 Australia; 2 Maclean District Hospital, Maclean, Australia

**Keywords:** Contralaterality, Optic primordium, Primordial eye field, Optic chiasm, Inversation, Chiasmation, Cyclopean, Translocation, Retinal progenitor cells

## Abstract

The primordial eye field of the vertebrate embryo is a single entity of retinal progenitor cells spanning the anterior neural plate before bifurcating to form bilateral optic vesicles. Here we review fate mapping data from zebrafish suggesting that prior to evagination of the optic vesicles the eye field may undergo a Maypole-plait migration of progenitor cells through the midline influenced by the anteriorly subducting diencephalon. Such an enigmatic translocation of scaffolding progenitors could have evolutionary significance if pointing, by way of homology, to an ancient mechanism for transition of the single eye field in chordates to contralateral eye fields in vertebrates.

## Background

The vertebrate forebrain (prosencephalon), with its rostral telencephalon and caudal diencephalon, exhibits a cluster of four neuroanatomic anomalies in all species. The evolutionary origins of hemisphericity, contralaterality, optic chiasm, and retinal inversion, continue to defy plausible explanation. If the eye as a neural budding of the light-sensitive diencephalon is central to the development of all, exposure of the enigmatic processes that transitioned the single eye spot of chordates to the paired eyes of vertebrates might explain these anomalies. If cogent, this explanation would clarify embryological homologies, enhancing potential developmental and medical implications.

Early in vertebrate gastrulation the neural plate is elongated by convergent extension [65] where laterally placed progenitor cells move toward the midline, intercalating into the midline [36, 37, 69]. This process of neurulation, which characterises the tubular folding of most of the neural plate to form the neural tube [9, 24] is a defining developmental process in vertebrates. The distinctive anterior of the neural plate (ANP) where the eye and forebrain develop, does not undergo this same neurulation as once thought [3, 23]. Its development begins earlier than neurulation, largely as a single coherent field of retinal progenitor cells (RPCs) that contracts from an early broad field to then bifurcate and evaginate to form bilateral optic vesicles [1, 2, 4, 15, 23, 31, 33, 46, 53, 64, 76, 80].

Contraction of the eye field (EF) follows an initial period of expansion characterised by proliferation of RPCs as gastrulation proceeds, with RPCs and their progeny migrating individually [71]. This contraction involves a ‘roll over’ migration of lateral RPCs over medial RPCs toward the midline [15, 64]. However, at the midline where RPCs are then observed to dive ventrally before apparently moving back laterally toward the periphery, there is condensed mixing of RPCs where some are lost to the contralateral EF [15, 64]. Further clarification is required of this moment of freneticism where large numbers of proliferated cells are condensed into a narrow midline field in a crush that briefly brings together cells that were furthest apart on opposite sides of the EF [15, 64]. This peak moment of midline invasion puts pressures on observation, decreasing confidence in the limits of imaging technologies and tests our abilities to observe precise continuity in single-cell lineages. What numbers and percentages of cells, for example, cross the midline and fail to return to their side of origin?

Historically, the conception that the EF of vertebrate embryos might actually be single before resolving to bilateral optic vesicles was intimately related to the discovery of midline crossings of RPCs [76, 80]. This discovery was inspired by the recognition that the human fetal abnormality *alobar holoprosencephaly* (cyclopia) is common across all vertebrate species [28, 45] and that the anomaly probably results from failure of a single EF to divide [5, 14, 28, 51, 76]. The most convincing evidence supporting this hypothesis came from a series of fate mapping studies late last century that employed highly specific individual cell-labelling techniques [2, 33, 76, 80] revealing that Nodal-deficient mutants fail to separate the EF, thereby exhibiting cyclopia [17, 26, 27, 66].

In wild-type zebrafish however, some RPCs and their progeny translocated across the midline to the contralateral optic vesicle, an observation that was used as primary evidence to argue that the EF was therefore singular prior to bifurcation [2, 76, 80], a view that is now the consensus. Another unrelated mixing of cells across the midline takes place in the remaining caudal aspect of the neural plate where a process called ‘mirror-symmetric divisions’ [74] create polar opposite clones which migrate to either side of the midline triggering formation of both neural rod midline and lumen.

## Quantifying the translocation

In 1995 a landmark single-cell fate mapping study of the EF in the zebrafish *Danio rerio,* while noting substantial translocation, did not explore any further significance of this translocation because of the more fundamental question at that time relating to whether cyclopia was a consequence of a single EF failing to divide or of two eye fields merging to become one. The incidental finding in this study that RPCs crossed the midline helped establish the general case for a prior single EF [80] but otherwise remained unexplored.

**Fig. 1:**
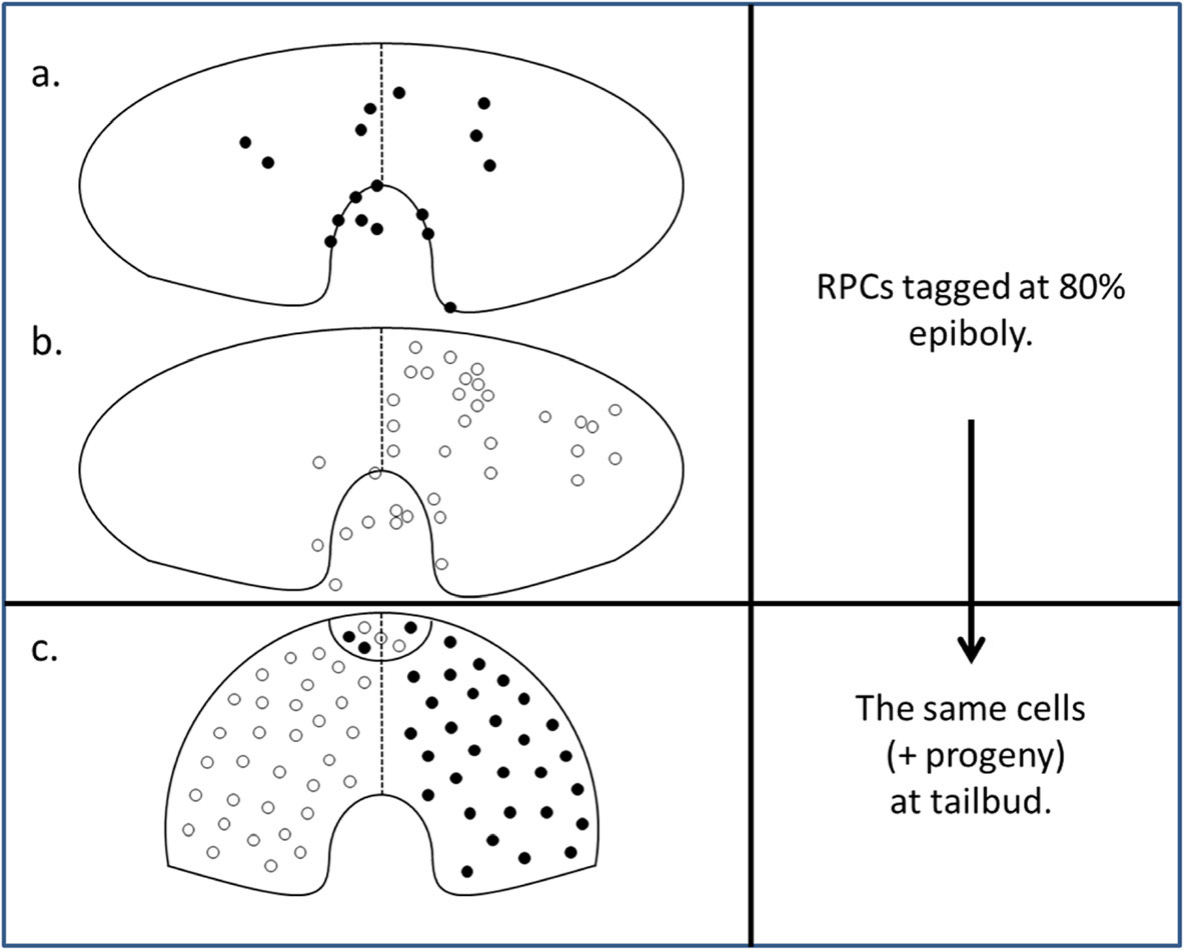
**a**, **b**, **c** Evidence for a translocating Optic Primordium. Cartoons adapted from previously published data [76]. Dorsal views of the ANP of wild-type zebrafish embryos (*Danio rerio*). Anterior to the top. The outline shows the limit of the EF (and *Opl* gene expression zone) and posterior indentation by the diencephalic *‘mar’* expression border subducting anteriorly. **a**, **b** Single RPCs were randomly labelled with lineage tracer (a mixture of rhodamine dextran and fluorescein dextran) at 80% epiboly. RPCs that matured in the right optic vesicle (**c**) are drawn here as dark circles, those that matured in the left optic vesicle (**c**) as clear circles. At 80% epiboly up to 30% of RPCs may have crossed the midline while the remaining 70% do so by tailbud. **c** at tailbud all RPCs (and progeny) are accounted for in optic vesicles as shown including within the presumptive optic stalk/chiasm (Maypole distribution). Cell numbers and locations in cartoon (**c**) are not intended to be precise

Four years later the most specific fate mapping study ever performed for single cell-lineage in the zebrafish EF was published just prior to the abandonment of this very specific technique in favour of mass-cell microscopy [76]. This study presented a detailed set of data that allows significant interpretation beyond the aims of the study itself and is here condensed and adapted in Fig. 1. Essentially, to show that the EF was a homogenous single set of RPCs prior to the anterior movement of the diencephalon that was thought to bifurcate the EF, single RPCs were injected with tracer dye at 80% epiboly and observed for fates at tailbud stage by sectioning the specimen. This was done repeatedly, one cell per specimen, and a control specimen, using a grid to plot positions of each cell labelled. The finding that a significant quantity of RPCs mingled across the midline to contribute to the contralateral optic vesicle was presented as support for the proposition that the EF was initially single but further significance was again not explored.

Closer scrutiny of the data as presented further shows that 70% of the RPCs labelled at 80% epiboly later crossed the midline to the contralateral optic vesicle and that some of the remaining 30% may already have crossed due to their proximity to the midline at 80% epiboly. Two outlying cells were omitted from our calculations as they were labelled as ending up on both sides. Another cell was excluded as it was located in the optic stalk. This left our total count at 53 remaining cells injected, with some minor uncertainty where red/blue labelling of a few cells in the original figure failed to clearly distinguish whether two single cells overlapped each other or a single cell contributed to progeny on both sides at tailbud. Our decision to include or exclude these few cells was based on a ten-fold magnification of the figure.

This proximity to the midline of the 30% suggests that if the cells had been injected earlier, say at 75% epiboly (8 h post fertilization - hpf) rather than at 80% epiboly, it is possible that more RPCs might have been found to originate on the side contralateral to their destinations. This would be consistent with a primordium that begins translocation across the midline prior to 80% epiboly, translocating up to 30% of its constituent cells by 80% epiboly, to be fully translocated at tailbud. (For stages of embryonic development see [38]).

The more recent studies on migration of RPCs [15, 64], rather than tracing a single cell and its clones, employed confocal time-lapse mass-cell microscopy techniques offering greater morphological specificity in terms of 3 and 4-dimensional organogenesis and stunning visual displays. These studies, in zebrafish and medaka fish respectively, unlike the previous study showed only minimal crossing of the midline by RPCs. However, it is not certain that these studies approximate the single-cell specificity of the earlier studies, especially at moments of intense congested agitation of cells such as at the midline during contraction of the EF. Compounding these uncertainties is the absence of verifiable quantification and dependence on visual time-lapse reconstructions from digital algorithms that are somewhat impervious to critical scrutiny.

**Fig. 2:**
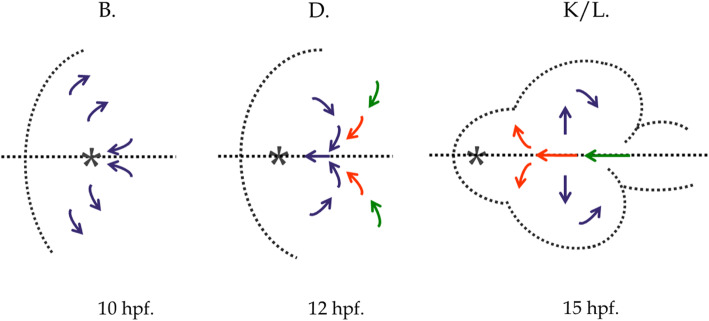
Three stages (B, D, K to L) of contraction and evagination of the zebrafish EF where the subducting neural keel is followed by a midline whorl of mixed cells from both sides. Adapted from Fig. 1. of [15]. Dorsal views, anterior to the left. Arrows show migration of progenitor cells: blue (eye field), red (optic stalk), green (diencephalon). Asterisks indicate anterior tip of neural keel formation (hypothalamus). HPF, hours post fertilization

Despite these limitations these studies made major advances, showing as never before the complex shape-changing over time of the developing EF. In particular they showed that prior to neurulation the EF begins posterior contraction toward a focus at the anterior tip of the hypothalamic anlagen (ventral diencephalon), the anterior limit of the neural keel (Fig. 2). As the keel subducts anteriorly beneath the EF posterior RPCs are drawn deep and anteriorly at the midline forming a transient whorl where RPCs from both sides move together along the midline. This focussed dynamic whorl is itself observed to advance anteriorly to eventually resolve at the site of the presumptive optic stalks as the optic vesicles evaginate [15].

The uncertainties discussed earlier as to whether these studies were capable of detecting RPCs crossing the midline appeared to be circumvented in a later study that employed Kaeda, a photoconvertible fluorescent protein introduced to the cytoplasm of the RPCs in zebrafish [32]. This was done to identify if any RPCs intercalate across the midline of the EF in the c–zipper fashion of the more caudal neural plate [7, 8, 22, 39, 74]. One half of the cells of the EF were converted from fluorescent green to red by UV illumination at commencement of imaging at 10.5 hpf. In this time-lapse series it was found that midline crossing of cells between 10.5 hpf and 13 hpf was rare.

This finding, however, is confounded by further uncertainties related to the timing of the observation period designed only to capture late cell developments rather than early migrations. The movie showing the photoconversion [32] depicts a single confocal plane, a transverse two-dimensional slice (plus time) through the developing forebrain beginning at 10.5 hpf. Timing is critical for our purposes because of the early anterior movement along the midline of the advancing whorl of RPCs. Contraction of the EF begins posteriorally at 8 hpf, anterior subduction of the hypothalamus immediately following [15]. Not only is the 10.5 hpf commencement of the movie two thirds through subduction late in this process, the transverse positioning of the confocal plane appears to be directed more posteriorly than anteriorly where contraction of the EF resolves early.

In summary, while the early ‘gold standard’ single-cell fate mapping studies point convincingly to significant midline translocation of RPCs, later mass-cell microscopy studies, while failing to confirm this significance, point to an intense transient mixing of RPCs at the midline of the posterior EF in a whorl which advances in tandem with the subducting hypothalamus. Behind its anterior advancement the EF is bisected, the whorl behaving like an advancing conduit, possibly for contralateral translocation of RPCs.

To reformulate: Based on presently established data from the most specific fate mapping study performed to date, at least 70% of RPCs that specify the EF domain and its future architecture will contribute to the contralateral optic vesicle. For this to happen, the RPCs that cross the midline must do so during peak contraction of the EF where they all appear to progressively mingle and dive ventrally in a whorl of congested midline hypermobility that advances from the posterior EF to the anterior EF, the latter being the site of the presumptive optic stalks. It is likely therefore, that the subsequent lateral movement of ventral RPCs previously thought to be of those returning to the ipsilateral EF from the midline between 8 and 12 hpf [15, 64] is in fact movement of RPCs that have crossed the midline from the contralateral EF via this dynamic conduit as the EF bisects.

## Eye field morphology in zebrafish

The mechanisms that drive the distinctive early development of the ANP, particularly the EF and optic chiasm, remain poorly analysed [23]. This is despite a more comprehensive understanding of the processes of eye and retinal development. The EF folds and bifurcates in a complex way, especially at the midline, but ultimately forms two pouches of RPCs that evaginate from the lateral epithelial walls of the diencephalon to give rise to the optic vesicles, the primordia of the eyes [32]. These evaginations are the consequence of RPCs migrating away from the midline after having converged to mix at the midline. While laterally moving cells have been shown to acquire either core or marginal attributes where mesenchymal core cells and marginal epithelial cells later undergo elongation and intercalation [32] the more intricate processes at the midline where the incipient optic chiasm forms are poorly defined.

During evagination the EF becomes patterned to give rise to a proximal future stalk domain and also a combined distal retinal pigment epithelium (RPE)-neural retinal (NR) domain [18, 29]. RPCs destined to form the eye continue to evaginate laterally toward the surface ectoderm [32, 41, 64] where, on contact, the optic vesicle invaginates to form the double-layered optic cup. The eye forms through a series of coordinated interactions between tissues of different origins: the retinal neuroepithelium, non-neural surface ectoderm, and a loose array of cells arising from both neural crest and mesoderm [19]. The inner layer of the optic cup is composed of prospective neural retinal cells and the outer layer composed of the primordium of the RPE [6, 19, 54]. The invagination process, which is associated with the development of the lens, leads to the formation of a transient opening along the ventral retina and optic stalk termed the choroid, or optic, fissure [68] which later fuses.

After positioning in the neural retina RPCs begin differentiating either to retinal ganglion cells (RGCs), horizontal cells, amacrine cells, cone cells, Müller cells, rod photoreceptors, or bipolar cells. While there are different stochastic models for the ordering of this process [44] the primacy of the RGC is well recognised. Once differentiated, RGCs begin a ‘retrograde axonal outreach’ back toward the midline, continuing centrally to cross the midline at the incipient optic chiasm before reaching the targeted contralateral ventral diencephalon.

## A prototypic optic chiasm

A general characteristic of vertebrates is the possession of bilateral eyes and an optic chiasm that connects the retinas to the forebrain. At the optic chiasm the left and right optic nerves cross, often completely, but in some groups of vertebrates particularly mammals, a fraction of the nerves remain uncrossed to form a semi-decussation. In the groups that mostly cross completely such as teleost fish, the decussation is mostly uncomplicated, left over right or right over left [34, 35]. The remainder of these groups have either a meshed chiasm or, as in the more primitive species of teleosts, a fused chiasm [57]. In the agnathan lamprey the mostly decussated chiasm contains a small ipsilateral component [11] and in the agnathan hagfish the chiasm is hidden within the hypothalamus [40]. While we cannot know the true structure of the optic chiasm in the first vertebrates the predominance of an uncomplicated complete decussation in most fish [79] allows that a simple crossing of one optic nerve over the other might be primitive for vertebrates. If so, the modern teleost might be a reasonable proxy for ascertaining primitive homologies.

Proximal prevertebrate chordates however, such as cephalochordates and urochordates, possess neither bilateral chambered eyes nor an optic chiasm. Cephalochordates though, possess a single medial eyespot which is now confidently regarded as the homologue of the vertebrate lateral eyes [43, 77]. Unfortunately, due to a paucity of both transitional fossils and extant progeny of transitional craniates, the mechanisms driving the evolutionary change from a single medial eye spot to bilateral eyes with a chiasm remain enigmatic.

## Toward a testable hypothesis for retino-forebrain evolution

**Fig. 3:**
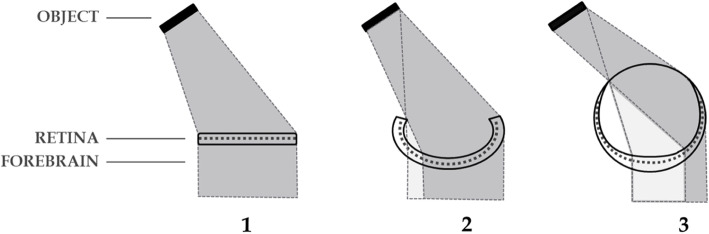
Inversation in prevertebrates. Progressive maturation of the cyclopean eye (1,2,3) by deepening of the retinal pit, favours contralateral inputs of light to the retina (dark shading) by exclusion of inputs ipsilaterally and centrally. This lateralization is maintained in the developing adnexal diencephalon. For further exposition of this concept see Loosemore [48]

While contralateral translocation of RPCs in the primordial eye field of vertebrates might seem counter-intuitive, as well as difficult to detect with time-lapse digital algorithms, there are historical precedents according to the Inversion Hypothesis [47–49] that suggest it may be imperative. This hypothesis postulates an initial 2-phase evolution of the eye and forebrain (retino-forebrain) in ancestral vertebrates punctuated by an ancient genome duplication resulting in a contralateral hemispherical forebrain and optic chiasm:

1. Inversation – the contralateralising of the non-hemispherical retino-forebrain of chordates by increasing selection of contralateral visual inputs due to maturation of the cyclopean eye. This theoretical framework, developed and published previously [48], is built particularly on the work of both Lacalli [42, 43] who showed that the single eye of the extant prevertebrate *amphioxus* (*Branchiostoma*) is homologous to the lateral eyes of vertebrates, and Nilsson and Pelger [59] who showed how the evolution of the primitive chambered eyes could have happened quickly by deepening of the retinal pits (Figs. 3 and 4).

**Fig. 4:**
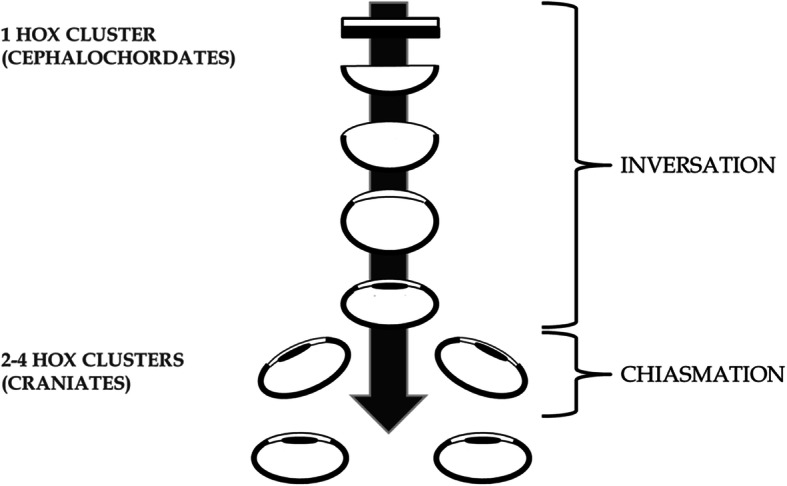
A genome duplication period coincides with duplication of the eye triggering new cyclopia-nulling genes such as *‘cyclops’* to initiate eye duplication. Arrow shows progression of time and eye maturation
2. Chiasmation – the later duplication of the single eye by a disruptive 3-vector developmental process; translocation, bifurcation, and invagination of the cyclopean eye field in craniates. This theoretical framework is constructed de novo as the most parsimonious way to duplicate a cyclopean eye while conserving the established contralateral retinofugal infrastructure of the primitive vertebrate forebrain (Figs. 4, 5 and 6). This event would likely have been triggered by new genes such as the Nodal-related ligand *‘cyclops’* (previously called Ndr2) following the genome duplication, acting homologously to today where in early embryogenesis ‘*cyclops*’ induces formation of bilateral optic vesicles from a single optic primordium [6, 25, 28, 63, 66, 76]. In this way “anterior expansion of a CNS ventral midline signaling system, involving cells specified by the ‘*cyclops*’ gene and increasing the size and complexity of the brain, might have been a key step during early evolution of vertebrates” [28].

**Fig. 5:**
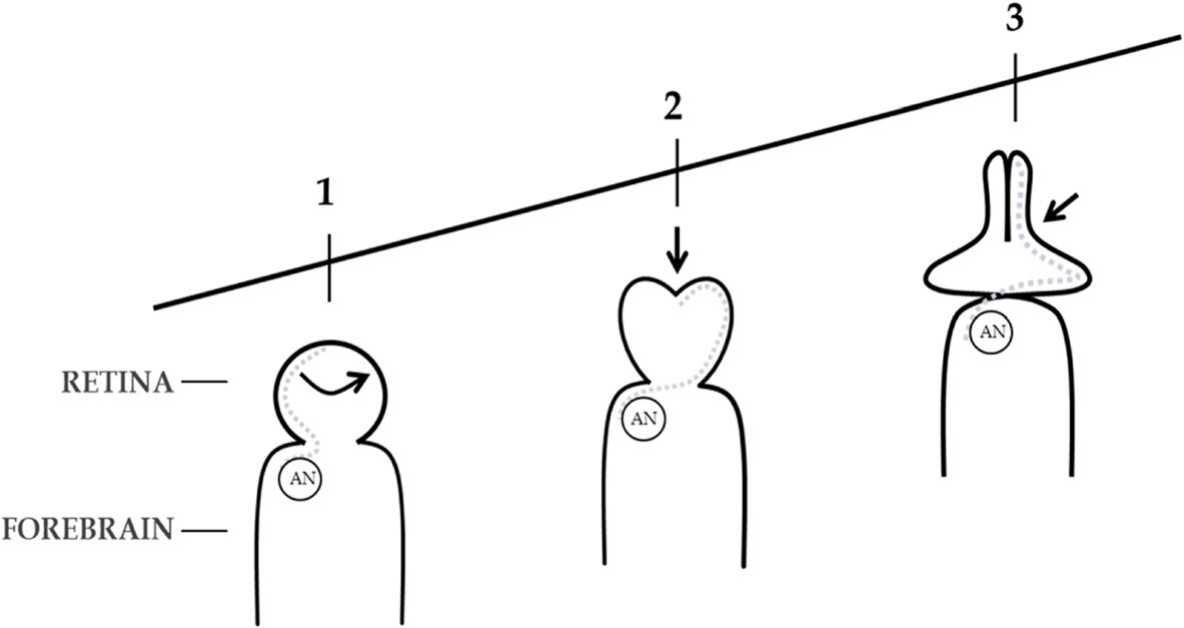
Chiasmation in the craniate embryo. Conceptual representation of EF duplication by the simultaneous 3-vector process: translocation, bifurcation, and invagination, resulting in bilateral eyes with retinal inversion and a chiasm. Anterior to the top. Dorsal representation. Arrows show vectors of change with vector 2 to be interpreted as midline rather than anterior. Dotted lines show the change in migration of RPCs across the midline. Final pathways reflect known retinofugal pathways to the putative anterior nucleus (AN) of the dorsal thalamus in extant primitive species

**Fig. 6:**
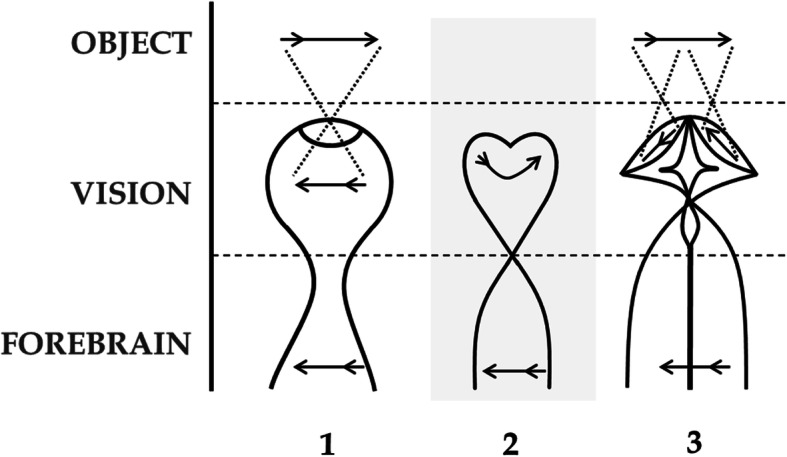
Eye duplication by chiasmation in craniates, with retinotopic conservation in the diencephalon. Contralateral forebrain infrastructure is conserved despite radical changes to retinal structures. Arrows show orientation of visual images. Anterior to the top. Dorsal representations. No 2 represents a conceptual (not actual) link between two adult phylogenies

Such a critical ancient chiasmation event, contributing as it would to the predatory power of the first vertebrates, should be identifiable in the very early morphological development of all vertebrate embryos today whereby:

- the EF should be single.
- it should translocate prior to bifurcation such that most RPCs (depending on the species) should cross the midline to mature in the contralateral optic vesicle.
- by this process the incipient optic chiasm should form and the retina invert.
- bifurcation of the EF should require the action of cyclopia-nulling genes which, if deactivated, will result in cyclopia.

Much of the above has been demonstrated experimentally in varying degrees in vertebrates such as zebrafish including now the apparent translocation of the EF leading to formation of the optic chiasm, the critical element of the proposed chiasmation process. As techniques for the observation and measurement of the origin and migration of embryonic cells (cell lineage) continue to advance we should expect techniques such as light sheet microscopy to eventually confirm and expand the findings already catalogued by past ‘gold standard’ techniques. If so, the finding that at least 70% of RPCs cross the midline to the contralateral optic vesicle should support the above hypothesis offering insights for:

- the single EF of vertebrate embryos (cyclopia in ancestral craniates).
- the contralaterality of the forebrain (Inversation by cyclopean eye maturation).
- the historical ‘point-of-origin’ of cyclopia-nulling genes (the Chiasmation event).
- the ‘mechanism-of-origin’ of the optic chiasm by Maypoling translocation of the EF (Chiasmation stage 1).
- the origin of eye duplication and forebrain hemisphericity (Chiasmation stage 2).
- the mechanism for inversion of the vertebrate retina (Chiasmation stage 3).

## Discussion

The suggestion that RPCs translocate en masse across the midline and the inference here that this might relate to the formation of the optic chiasm will be discussed below by way of question and response followed by a brief comment on the more peripheral topic of developmental retinal inversion.

### Should translocation of RPCs predetermine formation of the optic chiasm, and if so, by what mechanism might translocation and decussation be coupled?

The novel disruptive reorganisation of the retino-forebrain by the proposed chiasmation event is predicated on the introduction of new genetic, chemotactic, and mechanistic triggers, that while not yet fully elucidated are hypothesised to lead to the formation of the optic chiasm. However, the two distinct processes of translocation and decussation would appear to be coupled, not exactly in time, but almost in place, the former determining the latter.

While the sequential processes in the embryological development of the vertebrate optic vesicle, optic cup and retina are now quite well understood, the formative chiasmatic processes remain to be adequately described. This might, in part, be due to our incomplete understanding of the cellular milieu at the midline as the neural keel subducts anteriorly beneath the bifurcating EF to form bilateral incipient optic stalks and optic vesicles.

EF bifurcation follows anterior migration of the prechordal plate [46, 61] to move Nodal and *hedgehog*-secreting cells forward beneath the medial ANP. A consequent midline anteroventral ‘shearing’ process by the ventral diencephalon on the posterior EF begins separating the EF by initiating convergent migration of RPCs anteroventrally along, and possibly across, the midline of the EF. Our proposed major translocation of RPCs across the midline would appear during resolution of bifurcation to contribute to an early scaffold for the incipient chiasm by deposition of ‘remnant’ midline cells that might later cue RGC axons to decussate back across the midline. This idea of an early template for later growth of retinal axons was mooted by Easter et al. [13] and Sretavan et al. [72]. The latter claimed that in addition to neuroepithelial cells and glial cells, the presumptive chiasm contained a population of early generated neurons that express cell surface molecules capable of influencing retinal axon growth. According to Deiner and Sretavan [12], preceding arrival of RGC axons from the retina a partial incipient chiasm is in place at the ventral diencephalon (in the mouse embryo) which influences the completion of the chiasm. This principle is echoed by Ivanovitch et al. [32] who observed that the successful formation of the optic vesicles from embryonic stem cells is dependent upon a Laminin-rich Matrigel, a matrix protein that might provide an essential scaffold on which EF cells can organize.

Growth of RGCs begins in the retina and progresses centrally toward the presumptive optic chiasm after commitment of some of the multipotent progenitors to RGC fate. This happens during, or shortly after, the terminal cell division [56, 60, 62, 78]. Once RGC axons exit the eye they grow amongst the intrafascicular glial cells of the optic nerve [16, 70] with pioneer axons from the dorso-central retina navigating into the optic stalk ultimately traversing the midline to complete the formation of the optic chiasm.

As the RGC axons enter the midline region they encounter incipient optic chiasm neurons along its posterior boundary organized into an inverted V-shaped array [52, 55]. These neurons express the cell surface protein CD44 [72] and the SSEA-1 epitope [30, 52]. RGC axons grow through the chiasm region along the anterior edge of the CD44/SSEA-positive neuronal population which, in turn, facilitates the permissive decussation of RGC axons [52]. Experimental evidence also shows that these neurons are required for RGC axons to cross the chiasm midline [73].

### Does homology constrain developmental retinofugal options thereby necessitating an optic chiasm?

Map-like representation of sensory information is an evolutionarily conserved principle of brain organisation and function [50]. It follows that a species of pre-genome-duplication craniate would have had its forebrain retinotopy conserved in a post-duplication generation despite disruptive changes impacting the more malleable peripheral visual apparatus. An early retinal arrangement in cyclopean craniates that had achieved a dominant level of contralateral diencephalic representation should, if the retina was to be suddenly reorganised, have had the established retinotopy conserved in order that the species survived. Even after major disruption of the single median retina where each half resituated to the opposite side, this homologous constraint would have ensured that the relevant RGC axons crossed the midline to maintain retinotopic authenticity. This might feasibly have involved deposition and early differentiation of proximal midline progenitors at the site of the incipient optic chiasm following bifurcation of the eye field.

### Retinal inversion

The classic revision by Sarnat and Netsky [67] of Polyak’s 1957 theory of the development of the eye which convincingly described a very plausible pathway for development of the inverted retina no longer appears to fully satisfy the observations this century of a more complex unfolding of the ANP (For history of this theory see also [10]). While explanation for the inverted retina should continue to follow stepwise from the early neurulation of the ANP, the emerging understanding of the complex geometric unfolding of the single primordium should now constrain our options for describing the ontogenesis of retinal inversion.

## Conclusion

With expert opinion now shifting away from the traditional view that vertebrate eyes developed from an early intermediate bilateral stage exemplified by the rudimentary eyes in the extant hagfish agnathan [20] and toward a view favouring single eye duplication in protovertebrates [21, 58, 77] we await further palaeontological consideration like that of van der Brugghen [75] as to whether the earliest vertebrate fossils might now be seen to possess a frontal median eye. If such a finding transpires, Maypole-translocation of the EF in the vertebrate embryo would enhance the view that Inversation and Chiasmation might well inform the enigmatic intermediate processes for early vertebrate retino-forebrain evolution.

## Data Availability

Not applicable.

## Abbreviations

ANP: Anterior neural plate
EF: Primordial eye field
NR: Neural retina
RPC: Retinal progenitor cell
RPE: Retinal pigmented epithelium
